# The experience of persons with disabilities as beneficiaries of Ghana’s District Assemblies Common Fund

**DOI:** 10.1371/journal.pone.0297845

**Published:** 2024-04-11

**Authors:** Alimata Thelma Flora Abdul Karimu, Daniel M. Mont, Zachary A. Morris

**Affiliations:** 1 Department of Educational Foundations University of Cape Coast, Cape Coast, Ghana; 2 Center for Inclusive Policy, Washington, DC, United States of America; 3 Department of Social Welfare, Stony Brook University, Stony Brook, NY, United States of America; University of Environment and Sustainable Development, GHANA

## Abstract

Ghana’s Disability Fund aims to build the capacity of persons with disabilities, particularly those outside of formal employment, to engage in livelihood generation activities as a way to reduce poverty. The objective of this paper is to investigate the kind of knowledge that exists on the District Assemblies Common Fund program, understand the experience of beneficiaries when they access the program, and examine the benefits on beneficiaries’ livelihoods. The research consisted of five focus group discussions with 35 beneficiaries, key informant interviews with six member organizations of Ghana Federation of Disability Organizations, and interviews with eleven Disability Fund Management Committees members. This research found the experiences of beneficiaries on the program are varied yet the program on the whole has had a positive outcomes on their livelihoods. Persons with disabilities who participated in this study demonstrated knowledge of the program. Beneficiaries further described issues relating to the quality of purchased items, the procurement process, as well as reductions and changes to requested items. Disability-specific issues in accessing the funds were also noted. These limited the effectiveness of the Fund to meet its stated goals. The findings of the study can inform the Common Fund Secretariat efforts to improve the performance of the fund as well as the advocacy of the disability movement. The findings are also relevant to the design and implementation of other social protection programmes in low-and middle-income countries.

## Introduction

Ghana’s disability (DF) fund arose out of general efforts to reduce poverty. In 2001, the Government of Ghana accessed the Highly Indebted Poor Country initiative of the International Monetary Fund (IMF) to, among other objectives, address the high poverty levels among Ghanaians [1]. This resulted in the formulation and implementation of strategic frameworks called the Poverty Reduction Strategies (PRS). The first of the PRS documents was the Ghana Poverty Reduction Strategy I (GPRS, I) and the second, Growth and Poverty Reduction Strategy II (GPRS, II). These represented the development frameworks that were to guide the Government of Ghana after 2001 on economic growth, poverty reduction, and human development.

The GPRS I priority areas and key objectives did not focus on disability-specific and inclusive programs and policies. A critical examination of the GPRS I shows inclusive and disability-specific programmes were sparse and therefore yielded little benefit for persons with disabilities, [1]. However, moving forward, Chapter 8 of the GPRS 1 strategy called for special programmes for the vulnerable and excluded [1, 2], and recognized that persons with disabilities are a disadvantaged group with low levels of education and employment who need to be targeted by anti-poverty efforts [2]. Section 8.2.2 of the Chapter 8 of the GPRS 1 specifically highlighted the need for various programmes including community-based rehabilitation programmes; education programmes for physically and mentally challenged persons; and the provision of facilities and basic material support for schools for the blind and deaf [1].

Thus, the decision was made to introduce a disability specific programme to integrate persons with disabilities into mainstream production and employment. Government was not going to fully lead on disability-related agendas, but proposed partnership programs with nongovernmental organizations and acknowledged organizations of persons with disabilities (OPDs) as capacity building organizations [1].

As a result, the GPRS II addressed disability in its objectives, priorities, and strategies. For instance, transportation was recognised as a strategic support service within the agricultural and industrial sectors to be developed with the broad policy objective of recognizing access for persons with disabilities [3]. These gains in disability-inclusion in GPRS II are attributable to two interrelated developments. First, the fact that the Ghana Federation of Disability Organizations (GFD) was a member of the Cross Sectoral Planning Groups and whose consultative workshop in July 2005 provided input into the process [3]. Second, the demand on Ghana from international funding bodies to make national development expenditures focus on securing the welfare of the most handicapped and excluded elements of society ensured to a large extent disability-inclusion, where persons with disabilities were given monetary support through the District Assemblies Common Fund to enable them to operate small scale businesses. The objective of this paper is to investigate the kind of knowledge that exists on the District Assemblies Common Fund program, understand the experience of beneficiaries when they access the program, and examine the benefits on beneficiaries’ livelihoods.

## Background

### The District Assemblies Common Fund (DACF)

The DACF is derived from Article 252 of the Constitution of Ghana [4] and Act 455 (District Assemblies Common Fund Act) [5]. Basically, the DACF consists of a share of central government’s revenue provided to all Metropolitan, Municipal and District Assemblies (MMDAs) in Ghana for local development. It consists of funds not to fall below 5% of the total revenue the state (Common Fund) allots to the various Assemblies for development [5]. The Common Fund is therefore shared among the various assemblies through quarterly disbursements [5]. It comprises monies allocated to the Assemblies, other interests, and accumulated dividends from any investments.

### The disability fund’s original purpose

Embedded in Ghana’s social protection program, the DF was established to promote the development of the skills of persons with disabilities. Drawn from the DACF, the program aims to build the capacity of persons with disabilities, especially those outside of formal employment, to engage in livelihood generation activities as a way to reduce their poverty levels.

Since microfinance programmes and bank loans are largely inaccessible for persons with disabilities in the informal sector, the allocation of funds through the DACF system to persons with disabilities was intended to enable those outside of formal employment to participate in the labour market with government grants. This was seen to ameliorate conditions of extreme poverty and social deprivation among that particularly vulnerable group [1, 3].

Even though microfinance programs evolved and thrived in many developing countries as a means of poverty reduction, economic empowerment and employment avenues for the poor, there has been and continues to be a lot of evidence on barriers for persons with disabilities to access microfinance programs [6–8]. Specifically, Sarker [6] found persons with physical disabilities in Bangladesh encounter negative attitudes from microfinance staff, in addition to other significant barriers and exclusion, which are exacerbated by unaffordable credit conditions making it too hard for the ordinary person with a disability desiring entrepreneurial ventures to access them [6, 9–14]. Similarly, Nakayenga [15] also found persons with disabilities are discriminated against and excluded from microfinance programs. In this vein, Ghana’s DF was set up to give grants as startup capital especially to persons with disabilities without formal employment to equip them for livelihood generation as well as reduce the obstacles to getting loans from microfinance institutions and banks to enable participation and inclusion.

Arguably, the DF can be situated within the social model of disability where disability is complex and a consequence of the socio-economic and physical disadvantages faced by persons with disabilities and the negative perceptions impeding their full and equal participation in society [16, 17]. Therefore, the DF was focused on giving persons with disabilities the resources to overcome the barriers to economic participation, thus equalizing participation. The removal of financial barriers to ensure full participation of persons with disabilities in society fulfills aspects of the tenets of the United Nations Convention on the Rights of Persons with disabilities (CRPD) [18]. Among others, The CRPD [18] ensures for persons with disabilities the right to employment and work equally with others and have the opportunity to work freely in the labor market, with equal opportunities for self-employment, entrepreneurship, development of cooperatives and starting one’s own business (Article, 27.1, f).

At present, 3% of the DACF is set aside as the DF for persons with disabilities and has expanded from its original purpose. It is:

designed to support persons with disabilities to equip them in economic business ventures, assist them financially and provide them with working tools to empower them to become economically viable. The Fund also seeks to provide educational/training support for persons with disabilities as well as giving them medical/health support to make them functional in life” (Common Fund Secretariat Statement, personal conversation, 10^th^ March, 2022).

The goal of the Fund is to minimize poverty among all persons with disabilities particularly those outside the formal sector of employment, and to enhance their social image through dignified labor [19]. This goal comes with five objectives. These are to:

- Support the income generation activities of individual persons with disabilities as a means of economic empowerment.
- Provide educational support for children, students, and trainees with disabilities.
- Build the capacity of OPDs in the districts to enable them to advocate and assert their rights and undertake awareness raising and sensitization on disability issues.
- Provide medical support for individuals with disabilities, and
- Support persons with disabilities have access to technical aids and other assistive devices and equipment [19].

At present almost 8% (2,098,138) of Ghana’s population have differing degrees of difficulty in accomplishing activities or have some form of impairment. For persons 15 years and above with difficulty performing activities who are gainfully employed, 70.9% (seven out of every ten) are self-employed compared with 55.6% without any difficulty [20]. This is about 8% prevalence rate which has been shown to under-identify people with disabilities [21] so it is not surprising that this estimate is way below the WHO/World Bank estimate of global disability prevalence of 15% [22]. A study on the disability and poverty, though, still finds a significant difference in the rate of multidimensional poverty between people without disabilities in Ghana (44%) and those with disabilities (57%) and more recently 33.1% of persons with difficulty performing activities are multidimensionally poor in contrast to 29.1% of those without any difficulty [20, 23].

### Previous research on the disability fund

Previous studies of the DF suggest that while the program is beneficial, it has several operational issues. Agboga [24, and Agyemang 25] examined the contribution of the Disability Fund to the well-being of beneficiaries in selected MMDAs and found that the Fund was useful to beneficiaries. Fund recipients used funds to set up businesses or expanded existing ones, in addition to health and educational needs. Agyemang [25] further found 71% respondents indicating the Fund was useful for their livelihoods.

However, the disbursal of benefits was problematic. A study conducted by Ashiabi & Avea [26], investigating the absence of a disability measurement system in the disbursement of the Disability Fund in 13 districts in the Upper East Region, found the selection criteria was very skewed, when considering beneficiary characteristics, selection criteria and disbursement processes. They interviewed Disability Fund Management Committee (FMCs) members and recommended a social policy-oriented disability measurement system be adopted as an access criterion. Opoku et al., [27] found that few physically disabled, deaf, and visually impaired persons were actually able to access the fund, and that, there was insufficient funds to go round every applicant as well as saddled with disbursement delays. Their research focused on members of OPDs in four districts of the Northern Region. A study by Adamtey et al., [28] found that the Disability Fund did not adhere to the fund guidelines. In some instances, beneficiaries received the same amounts of funds for different purposes.

Moreover, the allocations were not sufficient to fulfill every request. This resulted in amounts been requested being halved or reduced to meet all requests at a time therefore rendering received funds insufficient for intended purpose [24, 25]. Additionally, Edusei et al., [29] in their study established that beneficiaries used the funds for the intended purposes but doubted whether the DF made real impact in the lives of beneficiaries since received amounts were too small.

Furthermore, Arkorful et al., [30], in assessing the policy efficacy and challenges of the fund in five regions of Ghana, found beneficiaries had limited information on the fund, faced access difficulties, disbursement delays and Metropolitan, Municipal and District Chief Executives (MMDCEs) interfering in the fund disbursement. They suggested policy restructuring and separating the fund management from political structures. Okrah [31] in examining the Common Fund policy in the Karaga district of the Northern Region also found delays in releasing funds from the Central Government.

Studies have argued that to improve the economic standing of persons with disabilities, the vulnerable among persons with disabilities need to be targeted for the DF [26, 32]. Nevertheless, the DF was not originally meant for every person with a disability, but to support those who are able to engage in economic ventures or already involved in income-generation activities with the aim of reducing poverty. Over the years, the scope has been broadened to cover medical, educational, and new proposals to cover the establishing businesses for mothers of severely disabled individuals.

### Contribution of this study

The research advances upon these prior studies by more deeply examining the lived experience of beneficiaries with accessing the fund and its impact, as well as gathering stakeholder information on the implementation and access challenges of administering the fund from some FMCs and leaders of GFD member organizations. Whereas most of the studies on the common fund have focused on the previous practice of handing out cash, this research looked at the current practice of purchasing items instead of handing out cash. Also worth noting, this research classified MMDAs into performing and nonperforming, to understand beneficiaries’ lived experience and implementation challenges within these differing policy settings. In furtherance, a detailed background to the establishment of the fund has been traced and foregrounded particularly distinguishing this research from all others.

## Methodology

A qualitative investigation was conducted in the Greater Accra Region of Ghana to gather information on the kind of knowledge that exists on the DF component of the DACF Program, ascertain the experience of beneficiaries when they access the Program and assess benefits of the program for beneficiaries. Through purposive sampling, beneficiaries, FMC members and leaders of OPDs were selected. The purpose of the qualitative study was to understand the full range of issues that people benefiting from the fund have in the Greater Accra Region. The Greater Accra Region was selected for this research since it is the only region in Ghana with the greatest number of Metropolitan and Municipal Assemblies. It has two Metropolitan, 23 Municipal areas and only four District Assemblies thereby making it one of the regions likely to receive significant funds from the Common Fund.

### Data collection procedure

A total of eleven Assemblies comprising one Metropolitan, nine Municipal Assemblies and one District were selected for this research considering rural and urban settlements. The Assemblies were: Tema Metropolitan Assembly, Ashaiman, Ayawaso West, Ga North, Kpone Katamanso, Korle Klottey, Krowor, La Dade-Kotopon, Okai Kwei North, Weija-Gbawe Municipal Assemblies and Ningo-Prampram District. With information from GFD member organizations, all MMDAs in the Grater Accra Region were classified into performing and nonperforming assemblies.

An MMDA was classified as a performing MMDA if they:

Often make Disability Funds available for beneficiaries, social workers and MMDCEs welcoming of persons with disabilities

Demonstrate the will to explain Fund-related issues and deferentially engage beneficiaries

Involve both OPDs and National Council on Persons with Disabilities (NCPD) representatives, enabling their effective participation in all FMCs meetings

Report regularly on the program to NCPD.

Eight Assemblies were selected from the performing group and three from the nonperforming Assemblies group (Table 1 below shows the Assembly classifications).

**Table 1:** Performing and nonperforming MMDAs.

Performing Assemblies	Nonperforming Assemblies
**Tema Metro**	Accra Metro
**Ashaiman Municipal**	Ada East District
**Ayawaso Central Municipal**	Ledzokuku Municipal
**Ayawaso West Municipal**	La-Nkwantanang Municipal
**Ayawaso East Municipal**	La Dade-Kotopon Municipal
**Ga South Municipal**	Kpone Katamanso Municipal
**Ga Central Municipal**	Ga West Municipal
**Korle Klottey Municipal**	Ga North Municipal
**Krowor Municipal**	Adenta Municipal
**Okaikwei North Municipal**	Ablekuma West Municipal
**Tema West**	Ablekuma Central Municipal
**Weija-Gbawe Municipal**	Ablekuma North Municipal
**Ada West District**	
**Ningo-Prampram District**	
**Shai-Osudoku District**	

Note. Performing MMDAs refer to Assemblies that often make available the Disability Funds for beneficiaries and nonperforming Assemblies those making access for beneficiaries very difficult. Information on Ayawaso North, Ga central and Ga East Municipalities were not available to help categorize them. Source: Authors, based on study data.

Using key informant interviews and focus groups, this research examined:

[1] The kind of knowledge that exists on DACF among persons with disabilities,

[2] the experience of beneficiaries when they access the Fund and

[3] the benefits of the program on beneficiaries’ livelihoods.

### Instruments and participants

#### Interviews

Key informant interviews were held with six leaders of GFD member organizations, namely: Ghana Association of Persons with Albinism, Ghana Blind Union, Ghana National Association of the Deaf, GFD representative, Sharecare Ghana and a sixth OPD that did not want to be identified But have members who have benefited from the DACF. In addition, eleven FMCs members from the eleven Assemblies were also interviewed. The Common Fund Secretariat was also contacted for interview. They however declined the interview and instead provided reports and other secondary information which have been incorporated in this paper. The GFD member organisations and FMC members will be referred to as ‘experts’ in this paper.

Key informants were chosen to include those directly responsible for the implementation and management of the Fund at the MMDAs levels. Leaders of OPDs who have traditionally led the advocacy around the DF were interviewed. Key Informants selection criteria at the MMDAs level included those directly handling the application process, those selecting beneficiaries, and those making allocation and disbursement decisions. Through purposive sampling participants were selected based on their involvement in either the implementation and/or management of the DF and leadership of OPDs that have traditionally led advocacy on and around the DACF.

#### Focus groups

Five focus group discussions (FGDs) were conducted with 35 selected Fund beneficiaries of which 19 participants were males and 16 females. Three of the FGDs were organized in Municipal Assemblies and one in a District Assembly. Additionally, three of the FGDs (two Municipal and the District Assembly) comprised participants with various impairments, the fourth, only persons with physical disabilities and the fifth, consisted persons with hearing impairment from various MMDAs. To ensure that participants from the same disability groups were not overrepresented in the focus groups, lists of Fund beneficiaries were obtained from the MMDAs and the GFD representatives on the FMCs and those beneficiary lists were used to populate the focus groups. Focus groups were set up to include people with albinism, hearing impairment, Muscular sclerosis, physical disability, cerebral palsy and visual impairment. Beneficiaries who were successful with the Fund and actively engaged in livelihood generation activities, beneficiaries who were not successful or have yet to begin any livelihood generation activities, and applicants on the waiting lists were all included. Beneficiaries who were not associated with any disability groups were also included, because of the concern that only including people from disability groups would populate the focus groups only with people with the most support in accessing the funds. FGD participants included three persons with albinism, four persons with visual impairment, two persons with muscular sclerosis, two persons with cerebral palsy, nine persons with hearing impairment and 15 with other forms of physical disabilities. Out of these, four persons were on the waiting list and three beneficiaries did not belong to any disability association at the time.

Each of the four focus groups were 90 minutes long except for the one with persons with hearing impairment which lasted 125 minutes to accommodate interpretation time. On average, there were seven participants in each group. Interview and focus groups guides were used to standardize the information gathered among the various groups. Interviews were conducted in-person and online. Both interviews and focus group discussions were recorded, and key informants compensated for their data costs and for participants, transportation.

*Ethical considerations*. Letters detailing the purpose and objectives of the research were sent to the selected MMDAs and the GFD to seek permission to interview key informants. Written informed consents were taken from key informants before the interviews. Informants were requested to sign, thumbprint or grant consent for their assistants to write their initials on the written informed consent form. We emphasized that participation was voluntary, they were not obliged to answer questions they did not want to, and that no one was going to be identified [33].

There was no intervention or experimentation conducted as part of this analysis, only the participation in a focus group or interview. Before all those interactions, the purpose and use of the data was explained, as were assurances of confidentiality. Informed consent was obtained from all the participants, and no identifying information is included in this paper except direct quotations.

### Data analysis

Thematic analysis was undertaken to ascertain and understand access challenges faced by beneficiaries and their representative organizations, and implementation challenges. Throughout this paper, we alter actual initials of beneficiaries and use the alphabet “E” representing Expert with numerals to refer to participants in the key informant interviews. The interviews and FGDs were coded using Microsoft Excel. In the analysis process, we read thoroughly to familiarize ourselves with the transcripts, and divided the data into themes. Following the thematic analysis framework outlined by Braun and Clarke [34], initial codes were generated as we familiarised ourselves with the transcripts and developed themes. The codes and themes were further discussed among the researchers to fine-tune the final themes reported on below.

### Findings on people’s experience with the disability fund

#### Knowledge of the program

Persons with disabilities who participated in this study demonstrated significant knowledge of the existence of the program, the application process and following up on their applications. Knowledge of the program was gained in meetings of their various representative organizations, district sensitization meetings normally organized by the Community Development and Social Welfare Departments of the MMDAs, GFD outreach programs and workshops, assembly members and the electronic media.

As a result of the involvement of assembly members in disseminating information and civil society groups advocacy around the DACF for persons with disabilities, the general public is now aware of the program which helps persons with disabilities to access the program:

I used to sell things with someone and one day someone came to buy items and asked if I know that the government has support for persons like me? I said no, so the person introduced me to the assembly. (AA).

#### Application process and follow-up

Closely related to beneficiaries’ awareness of the program is knowledge about the application process and follow-up, both of which beneficiaries were very familiar. Participants indicated that to apply for the funds, one needed to either write a letter or fill an assessment form from one’s MMDA. The assessment forms often have two sections: the first being personal information and the second, the purpose of funds being requested.

The application requires the applicant to indicate their name, contact number, house address, and then write whatever they are seeking for. The fund administrators don’t examine the construction of sentences and the like, just what the applicant is requesting. (TOR). For those who cannot read and write, the Social Welfare officers engage applicants and assist with the writing.

#### Quality of purchased items

In 2018, Fund operations changed from distributing cash to in-kind items or equipment. This directive was a response to beneficiaries misapplying requested funds and going back to the assemblies for more support (Common Fund Secretariat Statement, personal conversation, 10^th^ March, 2022). However, other forms of challenges emerged affecting beneficiary experience. One of these is the quality of purchased items. Beneficiaries reported that sometimes the items or equipment they received, even though new, have been of very low quality or “not durable.” Most beneficiaries interviewed expressed great disappointment with what they received:

I also wanted a mixer. They called me that my item had been bought. But, when I collected and used it, then it makes sh-sh-sh noises. So as for that one, I have put it aside and I am still using my old one. I could not use that at all. You see, there is a smaller version of the mixer which is very good and that is what I wanted and even what they bought for me was too expensive. The one that I am telling you is very good, is not as expensive as what they bought for me. (GG).

I requested for drilling machine and sandpaper machine and they all broke down. Oh, when I even started using it, it didn’t last beyond one week it got broken. They didn’t buy quality products for me. It was something funny, it was just like a toy to play with. It wasn’t strong machine. (LT).

There was no beneficiary who did not have anything to say about the quality of purchased items. Even with those who were lucky enough to have received something good, knew others who had items that did not last.

It is true. I know a man who is a carpenter who received a chisel and the day he used it, there and then, it broke down. That first day, it blew off. But when he took it to the assembly they did not change it for him and he is still waiting for his replacement. (FM).

I know somebody who also received canopy to be renting out. The day the person started using the canopy, when the wind blew a little, all came down. The canopy stand all broke down. (TOR).

These beneficiary experiences were further corroborated by the OPDs leaders, and some FMC members interviewed. They had begun receiving complaints from some beneficiaries on the quality of items:

Even in relation to the purchasing of the items that some of us seem to be happy about is the quality of the items or the durability of the items purchased. So, if I said I needed a printer, a refrigerator or some capital intensive item, one of the complaints currently is that, they receive the item, but the item is of inferior quality, does not last or stand the test of time once they begin to put them to use. (E3) “S1 Table”.

Those things that the persons with disabilities need, it’s given to the procurement people, it’s like they want to buy the cheaper things. If the person is not able to tell the exact brand he or she wants, that means that person will be given any inferior item. So now we’ve let them know if you want may be a laptop, write the brand that you want. Maybe I want this I want that, if not, they will give you an inferior thing. (E8) “S1 Table”.

#### Procurement issues

Another aspect of the quality challenge that came up in both the key informant interviews and the focus group discussions is the procurement process. When items and equipment go through procurement, they end up being too expensive. For example, ordinarily a freezer that should cost about GH₵2,500 would cost between GH₵3,500 or GH₵4,500 when it goes through assembly procurement procedures, further affecting beneficiary numbers and the quality of items. Expanding on this, experts and beneficiaries emphasized:

I think our major problem is the procurement because if I want to buy a freezer for someone, if I walk to the market to buy, it would be like GH₵1,400 or GH₵1,500. But then when it goes through procurements, then that means it is increasing to somewhere GH₵2,500 or GH₵3,600 which makes it very expensive. (E2). (See “S1 Table” for details).

Assemblies’ procured items were often highly priced and of low quality. When asked why Assembly purchased items were so expensive, an OPD leader on an FMC explained it was due to taxes:

When we question these things, they tell us that the procurement process makes the thing more expensive. For example, a wheelchair that should ordinarily cost 500 can cost 800 or more because they add certain taxes and the like. (UA). We also found Social Welfare Directors who are part of the FMCs interviewing and verting potential beneficiaries and their applications were not involved in the procurement. This results in people in charge of the procurement missing out on expert knowledge and insights when purchasing the items:

It is still with the procurement aspect. Because if we give out the list of items they need then that’s all. We have to sit aside and wait for the assembly to purchase. So we don’t have any say in the items they buy or whatever thing they will buy for the people; we don’t have any say (E8).

Additionally, some purchased items could not be used because they did not meet beneficiaries’ specific accessibility needs:

Later, some who also requested containers got booths. How can you move about in a small booth as a wheelchair user? How do you keep your items in that small enclosure? So, some people left theirs. About three or four were never claimed. The persons can’t use those. (UA).

Many people have got items they didn’t like or can’t even use. I went to the Social Welfare Director with TFA. To ask why we are sometimes given bad things like that. The Director said she has no hand in the purchasing of the items. But that there is a supplier who receives the cheque to purchase the various items. (SE).

There were reductions to requested items, equipment, and funds as well as not receiving the exact requested items. To reach many beneficiaries at a time, requested items were sometimes slashed. For example, some beneficiaries requested large poly tanks to retail water but received very small poly water tanks which do not help their work at all. Beneficiaries were very angry at receiving items and equipment short of expectations. Some beneficiaries also reported requesting 30 bags of maize for their corn dough business and ended up with 20 or 10 bags. The worrying aspect for the beneficiaries was, they were asked to sign for more bags of maize than they received. This we found was attributable to the procurement process as an Expert explained:

Because if somebody says they want to trade in provisions, or something else, you can’t use 1,000 Cedis, 1,000 of what provisions? What can it buy? You see, these things are going through procurement, so by the time they come, it would reduce and be like 400 Cedis worth of things. So 2,000 can be allocated to you but you will end up receiving items worth 400 Cedis (E12).

Other beneficiaries also reported getting freezers and items that were too small for their purpose. They wanted to store and sell fish, but then, very small fridges or deep freezers were bought for them. This resulted in some beneficiaries having to step down on their intended purpose of applying for the Fund:

I applied for a deep freezer and some cash to enable me buy chicken and tilapia to sell. Later when I was called to come for my items, I only received the fridge. When I asked them for the money, they said I should try and get the money for the tilapia and chicken. But I don’t have that kind of money for those things. I later decided to use the fridge to rather sell ice water. If I had received the money for the tilapia and chicken business would have been better than this ice water that I am selling now. (AM).

This woman also wanted to stock her shop and the following is what she got:

I applied for the DACF, and I requested for GH₵3000. And I wanted to sell diapers and sanitary pads. Yes, this was my proposal. And they invited me to come. When I got there, they just put one pack of baby diapers, sanitary pads, some few things one pack each and then they added GH₵100 to it for me. So, I asked them what am I going to do with this? These are things that I am going to sell but they are not something that I am going to use for myself. I refused to take them. But one of the officers of the Department of Social Welfare pleaded with me to take the items and that later she will call me to come back for a discussion or a top up. So later when I did the follow-up I went there and they gave me GH₵700. And rather they wanted publicity, they took a picture of me and then announced that yes, they have set me up and all that. Out of the GH₵3000 that I requested. (BB).

Again, in certain circumstances, the exact requested items were not purchased.

I have heard a lot of issues because I am the Regional Secretary, and it has been a challenge. If someone says I want a fridge may be the person has started from somewhere and then you change what they want. You know the fridges we have sizes, if I want 20 feet or whatever, they will go and change and buy me about 10 feet. It doesn’t help. Always you don’t send applications for them to give you exactly what you want to set you up. Always, they have to change what you send to them. The responses are also not fair. Sometimes they will tell you that, if you don’t like it leave it. (SJ).

This research found the FMCs normally want funds to reach as many beneficiaries as possible hence the reductions to requested items or funds. But beneficiaries in these focus groups preferred to receive significant help to make them self-reliant for long periods as echoed by this beneficiary:

When we went for the interview, they said we should use the funds for work and to stop begging. And the funds when they give us, you have to plan that this type of work is what I would want to do. But when you tell them the exact funds or thing you need for your business, they will not give you, but they will give you something small. (OC).

#### Disparity in allocations and lack of transportation support in some assemblies

There is also a disparity in terms of the items that beneficiaries received. For instance, applicants who applied through or were recommended by MMDCEs, GFD representatives, Assembly members and FMC members were more likely to receive the exact requested items, including larger or more durable equipment, and multiple times than those who apply as ordinary persons. For example, if six people request for 2500-litter poly water tank, and two were not recommended or did not know any significant person, they would receive 800-litter poly water tank instead of the 2500-litter capacity originally requested. Speaking to this, an expert bemoaned:

So if there is a favoritism then it’s not from our office but from the GFD rep. Sometimes you get them interfering. This person, this person, or that person has this problem it’s urgent, do you deny the person the chance? So you see? But to the applicants they think that we are here and we have favorites. We are always taking care of everybody. But favoritisms don’t come from us. And then the political interference. You are here and then the Chief Executive will send, ten people. When you look at the ten people, they have all benefited before. May be last year, then, because the list is coming from Chief Executive, sister, what would you do? Would you tell him that you won’t take care of the people? So you see, these are some of the set backs (E11).

This practice was common in both performing and nonperforming assemblies and was not specific to any political party. Similarly, Ashiabi & Avea [26], Banks et al., [35], and Banks and colleagues [36], all assert that biases and differential treatments can affect program effectiveness and reach.

Also, there is no uniformity in the allocation process for example. Whereas some MMDAs assist beneficiaries with transportation cost when they claim their items, others do not. This created extra financial burden especially for those receiving bulky items and equipment. Failure to take into consideration additional costs and barriers experienced by persons with disabilities have also been noted by Walsham and colleagues [37].

#### Disability-specific issues

Certain types of disabilities such as albinism were not recognized as a disability, making it harder for persons with albinism to access the Fund in some Assemblies. This expert asserted:

Yeah, for persons with albinism, it is not very easy for them to access in many of the districts. The reason being that some people still do not believe that persons with albinism are persons with disabilities. So, most times, the problem that comes out is that the money is for persons with disability but not for persons with albinism. So, there have been situations where we have to engage some Social Welfare Directors, who have been in the social work field for about 26 years. And they didn’t seem to understand the category of disabilities that they were dealing with. (E4).

Furthermore, people who are deaf experienced communication difficulties with FMCs and MMDAs staff. There were often no Sign Language Interpreters which resulted in Assembly staff either resorting to writing or using gestures which did not promote meaningful communication for persons who are deaf:

Communication is not easy it is difficult because for me when I went there, they didn’t invite a sign language interpreter to communicate with me. They asked me to write so they were communicating with me through writing. (MM);

You know, if you are talking in your own dialect, you see how you can express yourself. But then for people who are deaf, we have no speech. Many people also don’t understand the sign language. So even when you go to meetings, and you don’t even get a professional sign language interpreter, to be able to articulate the views of persons who are deaf to participant is difficult. You find out that a person who is deaf still has those problems of communication, and information. (E5).

## Benefits of the fund

Notwithstanding a number of the access challenges and some disappointing experiences of beneficiaries, they all acknowledged that the programme helped to promote their inclusion into their community and family life, enhanced their dignity and feeling of being valued in the household and community at large. The Fund has assisted beneficiaries to establish small-scale businesses, acquire skills and education, access critical health services, assistive devices among others. For instance beneficiaries with physical disabilities who received funds and established sowing businesses shared how they had to struggle and walk long distances to knit clients’ clothes but this has been made easy since they now have their own machines. Moreover, the knitting machines bring in additional income since they knit for outsiders also:

At first after sewing, I had to take the clothes to another place for the knitting to be done, and then I will pay. But now, I have my own machine. So as soon as I finish, I will just do the knitting. Oh yes, I do knitting for outsiders also. So it brings in additional income or some more coins. That knitting machine has helped me a lot. (TAR).

Some beneficiaries also use the opportunity of the fridges they get to add other essential food commodities for sale which increases their overall profit margins:

Me too, mine has helped me a lot, the fridge. I sell water, drinks, and the provisions also sell very well by the grace of God. Whatever happens, we get something to support ourselves, give some to the children for school, should they want water to drink, they get, and all that. It has helped us and our families value us. (AH).

For me, the fund has been useful. I have managed to purchase some of the tools myself and I’m using them in addition to the common fund ones that did not break. I am still doing my business. (LT).

I will say in general, it is good. For me also, I am still using my machine for my work, it has helped me in that regard. But if was possible, I would have applied for another machine again to enhance my work. (OE).

In terms of educational support, there was evidence to show that laptops had been purchased for some people. Also, other beneficiaries in the FGDs had their school fees paid and Experts in the KIIs attested to paying school fees and provided provisions for students and pupils.

The previous ones I received actually helped me, it helped me a lot. I was then learning craft at the Accra Rehab Centre, so I was taking to pay my school fees. (LLS).

We pay for the school children, we have two who have already completed Legon. So now we have one, I think she is in Level 200 now at Legon and another at University of Cape Coast. Then, we have the younger ones at Mampong School, and then Adjei Kwadjo and then the Ashaiman Schools. Then a few at Dzorwulu Special School too. (E11).

Even though we had categorized the MMDAs into performing and nonperforming, findings did not suggest performing MMDAs had less of the identified issues discussed in this section than the nonperforming assemblies. However beneficiaries in performing MMDAs were likely to have received more than one round of funding and also more likely to receive extra funds (albeit small) to cater for conveying purchased items. For example, in Weija-Gbaweh Municipal assembly, beneficiaries reported receiving GH₵50 as transportation for everybody who received items in 2022.

## Discussion

Ghana’s disability fund arose out of general efforts to reduce poverty among persons with disabilities without employment. In 2001, the Government of Ghana accessed the Highly Indebted Poor Country initiative of the IMF to address the high poverty levels among Ghanaians as well as roll out reform programs needed to restore macroeconomic stability [1]. As a result of this, development partners required Ghana to make national development expenditures focus on securing the welfare of the most handicapped and excluded people in society. The DF was therefore established for persons with disabilities to receive monetary support through the District Assemblies Common Fund to enable them to engage in livelihood generation.

This research investigated the lived experience of beneficiaries with accessing and impact of the fund; implementation and access challenges with administering the fund from some FMCs and leaders of GFD member organizations. Specifically, information was gathered on the kind of knowledge that exists on the Disability Fund component of the DACF Programme; ascertained the experience of beneficiaries when they access the Programme; and examined benefits on beneficiaries’ livelihoods. Consistent with some areas of findings of Opoku and colleagues [27], this research found FGD participants had knowledge of the existence of the program. Their sources of knowledge included assembly members, Social Welfare workers at GFD meetings and via the electronic media. Again, beneficiaries were aware and knew the application, submission, and follow-up processes.

The research further indicated how limited funding for the DF complicates the administration and effectiveness of the program. Concurring with Arkorful et al., [30], this research found that beneficiaries could not invest in sustainable ventures when they received only small amounts. It further found that beneficiaries who did not receive the exact requested item or funds had to step-down on their intended purpose. Beneficiaries preferred to receive substantial amounts that would make them self-sufficient for long periods and would thus help establish their income generating activities. From the perspective of FMC members, allocating reduced amounts of funds enabled more applicants to benefit from the program [25, 27, 29]. However, in the focus groups with beneficiaries, it was found that this dilution of the funds comes with a tradeoff of which limits the impact of the fund. This dilemma is indicative of the systemic challenges inherent to administering a fund with limited overall fiscal capacity to meet the high amounts of need in the community.

Despite the 2018 change aimed at improving the mode of fund receiving, where beneficiaries no longer get physical cash but items, this research has revealed a number of worrying issues. These include: low quality of purchased items; procurement issues; reductions to requested items, equipment, funds, and not receiving exact requested items; disparity in allocations and lack of transportation support in some assemblies; and disability-specific issues.

Among the people interviewed, beneficiaries who were recommended by MMDCEs, GFD representatives, Social Welfare officers and Assembly members were likely to receive durable or quality items than others. This experience aligns with literature highlighting political actors influencing Fund allocation decisions can result in differences in allocations Edusei and colleagues [29], Arkorful et al., [30] as well as effectiveness Banks et al., [35], and Banks et al., [36].

The benefits of fund on beneficiaries’ livelihood as evidenced in this paper is further consistent with Agyemang [25], and Edusei and colleagues [29]. We also found beneficiaries felt their sense of dignity was enhanced as a result of engaging in a livelihood generation activity and felt valued in their family and community life.

Finally, in both the performing and nonperforming Assemblies, all the challenges discussed above were common. However, in nonperforming Assemblies, beneficiaries had more problems with the quality of purchased items, did not receive additional funds as start up capital to enable them stock their fridges or shops, were required to bear the cost of transporting received items, did not receive any additional funds even when beneficiaries were doing well with already-received funds but needed capital injection to expand businesses, and the disbursement process did not include representatives of NCPD and GFD. Also, complaints of disparities in items received were prevalent in these Assemblies. Actually, in Assemblies where Chief Executives interact with and welcome persons with disabilities, beneficiaries reported having many disbursements than in those Assemblies where MMDCEs always seem busy and constantly referring them to the Social Welfare officers.

## Limitations

Importantly, this study is a qualitative study examining people’s experiences with accessing the Disability Fund of the DACF but could not interview beneficiaries from every MMDA in the Grater Accra Region. Similarly, not all FMC members were also interviewed as some declined our request. In addition this study does not include quantitative data as we wanted to get rich experience. Therefore our aim is not to generalize the findings but demonstrate the program effectiveness or the lack of it in this region.

## Recommendations

The findings presented herein support the need for greater funds dedicated to the Disability Fund to meet the high demand and improved procurement systems. Procurement officers of the MMDAs should be required to liaise with Social Welfare Directors who are involved in the needs assessment and vetting of potential beneficiaries before items and equipment are procured. A grievance procedure should be established for beneficiaries to easily lodge complaints when they receive inferior or inappropriate goods.

In further identifying the accessibility issues experienced by people who are deaf in accessing the fund, the findings suggest the need for sign language interpreters trained in MMDAs, which could be provided in collaboration with the Ghana National Association of the Deaf. A process should be developed where people who are deaf can request interpreters in advance when visiting MMDAs. The GFD and OPDs should further continue advocacy on the evolving nature of disability across the country to limit the incidences of some categories of disabled people, such as albinism, not being recognized by Social Welfare personnel and FMC members.

## Conclusion

Our goal in this study was to investigate the experiences of beneficiaries with accessing the Disability Fund and examine the benefits on beneficiaries’ livelihoods. A qualitative study was therefore conducted in the Greater Accra Region of Ghana to gather information on the kind of knowledge that exists on the Disability Fund component of the DACF Programme, ascertain the experience of beneficiaries when they access the Programme and assess benefits of programme for beneficiaries. We found the experiences of beneficiaries on the programme are varied yet on the whole the programme has had a positive outcome on their livelihoods. We, however, stress the importance of having the voice of persons with disabilities included in the design and evaluation of the program which is vital to advancing it’s objectives.

## Supporting information

S1 Table FGDs codes. (XLSX)

## References

[1] Ghana National Development Planning Commission. An Agenda for Growth and Prosperity (GPRS I) (2003–2005). Accra; 2003.

[2] Republic of Ghana. Country Review Report: Strengthening Efforts for the Eradication of Poverty and Hunger. The Annual Ministerial Review. Geneva, Switzerland; 2007.

[3] Ghana National Development Planning Commission. Growth and Poverty Reduction Strategy (GPRS II) (2006–2009): IMF Country Report 06/225. Accra; 2005.

[4] Constitution of the Republic of Ghana. Accra; 1992.

[5] Act 455, District Assemblies Common Fund Act. Republic of Ghana; 1993. https://ghanalegal.com/laws_subdomain/acts/id/119/district-assemblies-common-fund-act/#

[6] Sarker D. Experiences of people with physical disabilities when accessing microfinance services in Bangladesh: a qualitative study. European Journal of Disability Research. 2022;16(3): 41–55.

[7] Ranabahu N, Tanima FA. Disabled women entrepreneurs and microfinance: a road less travelled (for a reason)? Research Handbook on Disability and entrepreneurship. 2022;196–207.

[8] Mont D. Employment programs for people with disabilities in Low- and Middle-income countries. In: Heymann J., Stein M. A., and Moreno G., editors. Disability and Equity at Work. New York: Oxford University Press; 2013.

[9] Nuwagaba EL, Nakabugo M, Tumukunde M, Ngirabazunki E, Hartley S, Wade A. Accessibility to micro-finance services by people with disabilities in Bushenyi District, Uganda. Disability and Society, 2012;27(2): 175–190. doi: 10.1080/09687599.2011.644929

[10] Sarker D. Microfinance for disabled people: how it is contributing? Research Journal of Finance and Accounting. 2013;4(9): 118–125. doi: 10.7176/RJFA/4-9-118

[11] Sarker D. Inclusion of disabled people in microfinance institutions: where does Bangladesh stand? International Journal of Innovation and Economic Development. 2015;1(1): 70–82.

[12] Nuwagaba EL, Rule PN. An adult learning perspective on disability and microfinance: the case of Katureebe. African Journal of Disability. 2016;5(1): a215. doi: 10.4102/ajod.v5i1.215 28730047 PMC5433452

[13] Sarker D. Discrimination against people with disabilities in accessing microfinance. Alter-European Journal of Disability Research/Review. 2020;14(4): 318–328.

[14] Mulubiran T. Livelihood assets and strategies of people with disabilities in urban areas of Ethiopia. Scandinavian Journal of Disability Research. 2021; 23(1): 63–73.

[15] Nakayenga C. The determinants of financial accessibility among people with disabilities in microfinance institutions in Jinja municipality: a case study of pride microfinance Uganda limited Jinja branch [doctoral dissertation]. Makerer University; 2021.

[16] Oliver M. A sociology of disability or disablist sociology. In Barton Len, editor. Disability and Society: emerging issues and insights: Harlow, Essex: Addison Wesley Longman Limited; 1996. 18–42.

[17] World Health Organization (WHO). International Classification of Functioning, Disability and Health (ICF). 2017 http://www.who.int/classifications/icf/en/.

[18] United Nations Convention on the Rights of Persons with Disabilities (UNCRPD), A/RES/61/106. (December 13, 2006). https://www.un.org/development/desa/disabilities/convention-on-the-rights-of-persons-with-disabilities-html

[19] Ghana National Council on Persons with Disabilities (NCPD). Guidelines for disbursement and management of the disability component of the District Assemblies Common Fund. Accra; 2010.

[20] Ghana Statistical Service. 2021 Population and Housing Census iii: Population of Regions and Districts Report. 2022. https://statsghana.gov.gh/gssmain/fileUpload/pressrelease/2021%20PHC%20General%20Report%20Vol%203F_Difficulty%20in%20Performing%20Activities_final_161221.pdf

[21] Mont D. Measuring Disability Prevalence, Social Protection Discussion Paper No.0706, The World Bank; 2007.

[22] WHO, World Bank. World Report on Disability. 2011 https://www.who.int/teams/noncommunicable-diseases/sensory-functions-disability-and-rehabilitation/world-report-on-disability

[23] Disability Data Initiative. Disability Data Report. 2022 https://disabilitydata.ace.fordham.edu/twentyreport/disability-data-initiative-2022-report/

[24] Agboga RS. The contributions of the District’s Disability Common Fund to the well-being of beneficiaries in Ada East District [MA]. University of Ghana, Accra; 2015. http://ugspace.ug.edu.gh/handle/123456789/21814.

[25] Agyemang EF. The effect of the Disability Fund on individual livelihoods. A case study of persons with disabilities in the Atwima Nwabiagya District of the Ashanti Region of Ghana. (MSc). Kwame Nkrumah University of Science and Technology; 2015. http://ir.knust.edu.gh/handle/123456789/8282.

[26] Ashiabi EE, Avea AP. The absence of a disability measurement system in the disbursement of the District Assembly Common Fund for persons with disabilities in Ghana: How the most vulnerable are denied access. Review of Disability Studies; 2019; 15(4).

[27] Opoku MP, Nketsia W, Mprah WK. Extending social protection to persons with disabilities: exploring the accessibility and the impact of the disability fund on the lives of persons with disabilities in Ghana. Global Social Policy; 2018; 19(3).

[28] Adamtey R, Oduro CY, Braimah I. Implementation challenges of social protection policies in four districts in Ghana. The case of the District Assembly Common Fund meant for persons with disabilities. Legon Journal of the Humanities; 2018; 29(1). https://www.ajol.info/index.php/ljh/article/view/173263.

[29] Edusei AK, Adjei-Domfeh P, Mprah WK, Opoku MP, Badu E, Appiah CS. Assessing the impact and uses of the disability common fund among persons with disabilities in Kumasi Metropolis in Ghana. Review of Disability Studies; 2016; 12(4).

[30] Arkorful VE, Anokye R, Basiru I, Hammond A, Mohammed S, Micah VB. Social protection policy or a political largesse. Disability Fund efficacy assessment and roadblocks to sustainable development goals. International Journal of Public Administration; 2020; 43(15); 1271–1281.

[31] Okrah M. The control mechanisms for effective disbursement and utilization of the District Assemblies Common Fund in the Karaga District of Northern Ghana. Journal of Economics, Management and Trade; 2016; 11(4): 1–15.

[32] Naami A, Mikey-Iddrisu A. Empowering persons with disabilities to reduce poverty: a case study of Action on Disability and Development, Ghana. Journal of General Practice; 2013; 1(2) 100–113.

[33] Israel M, Hay I. Research ethics for social scientist: Between ethical conduct and regulatory compliance. Sage Publications Ltd; 2006.

[34] Braun V, Clarke V. Using thematic analysis in psychology. Qualitative Research in Psychology. 2006;3(2);pp. 77–101.

[35] Banks LM, Walsham M, Minh HV, Duong DTT, Mai VQ, Blanchet K et al. Access to social protection among people with disabilities: Evidence from Vietnam. International Social Security Review; 2019a; 72(1); pp. 59–82.

[36] Banks LM, Walsham M, Neupane S, Neupane S, Pradhananga Y, Maharjan M, et al. Access to social protection among people with disabilities: Mixed methods research from Tanahun, Nepal. European Journal of Development Research; 2019b; 31(4).

[37] Walsham M, Kuper H, Banks LM, Blanchet K. Social protection for people with disabilities in Africa and Asia: a review of programs for low-and Middle-income countries. Oxford Development Studies; 2019;47(1). doi: 10.1080/13600818.2018.1515903

